# CT and MRI Imaging of Theranostic Bimodal Fe_3_O_4_@Au NanoParticles in Tumor Bearing Mice

**DOI:** 10.3390/ijms24010070

**Published:** 2022-12-21

**Authors:** Alexey A. Lipengolts, Yulia A. Finogenova, Vsevolod A. Skribitsky, Kristina E. Shpakova, Adi Anaki, Menachem Motiei, Alevtina S. Semkina, Maxim A. Abakumov, Anna V. Smirnova, Elena Y. Grigorieva, Rachela Popovtzer

**Affiliations:** 1 N.N. Blokhin National Medical Research Center of Oncology, 115478 Moscow, Russia; 2 The Alexander Kofkin Faculty of Engineering, Bar-Ilan University, Ramat Gan 5290002, Israel; 3 Bar-Ilan Institute for Nanotechnology and Advanced Materials (BINA), Bar-Ilan University, Ramat Gan 5290002, Israel; 4 Department of Medical Nanobiotechnoilogy, N.I. Pirogov Russian National Research Medical University, 117997 Moscow, Russia

**Keywords:** nanoparticles, CT, MRI, contrast agent, tumor

## Abstract

Gold-containing nanoparticles are proven to be an effective radiosensitizer in the radiotherapy of tumors. Reliable imaging of nanoparticles in a tumor and surrounding normal tissues is crucial both for diagnostics and for nanoparticle application as radiosensitizers. The Fe_3_O_4_ core was introduced into gold nanoparticles to form a core/shell structure suitable for MRI imaging. The aim of this study was to assess the in vivo bimodal CT and MRI enhancement ability of novel core/shell Fe_3_O_4_@Au theranostic nanoparticles. Core/shell Fe_3_O_4_@Au nanoparticles were synthesized and coated with PEG and glucose. C57Bl/6 mice bearing Ca755 mammary adenocarcinoma tumors received intravenous injections of the nanoparticles. CT and MRI were performed at several timepoints between 5 and 102 min, and on day 17 post-injection. Core/shell Fe_3_O_4_@Au nanoparticles provided significant enhancement of the tumor and tumor blood vessels. Nanoparticles also accumulated in the liver and spleen and were retained in these organs for 17 days. Mice did not show any signs of toxicity over the study duration. These results indicate that theranostic bimodal Fe_3_O_4_@Au nanoparticles are non-toxic and serve as effective contrast agents both for CT and MRI diagnostics. These nanoparticles have potential for future biomedical applications in cancer diagnostics and beyond.

## 1. Introduction

Nanotechnology currently plays an important role in medical research. Unique physical and chemical properties of nanoparticles provide the opportunity to create multifunctional nanoplatforms that integrate therapeutic and diagnostic characteristics. Oncology is the most extensive field for the application of theranostic nanoparticles, since they can be used simultaneously in the diagnostics and therapy of cancer without alterating their pharmacologic nature.

Metal and metal oxide nanoparticles are being investigated as radiosensitizers for radiation therapy and contrast agents for diagnostic imaging. NBTXR3 and AGuIX are metal-containing nanoparticles undergoing clinical trials. NBTXR3 is a radioenhancer composed of hafnium oxide. NBTXR3 nanoparticles have demonstrated safety and therapeutic efficacy in combination with radiotherapy for locally advanced soft-tissue sarcoma [1]. After intratumoral injection, the zone of nanoparticles’ retention was clearly visible in CT [2].

AGuIX are sub-5 nm nanoparticles made of a polysiloxane matrix and gadolinium chelates. AGuIX was verified as a theranostic agent for radiotherapy of brain metastases. The biodistribution of AGuIX and its uptake value in the metastases were monitored by MRI [3,4]. Besides clinical trials, many preclinical in vivo studies of theranostic metal nanoparticles proving their efficacy are being performed [5,6,7].

Among metal nanoparticles, gold nanoparticles are considered to be the most effective dose-enhancing agent in radiotherapy due to the high atomic number (Z = 79). A number of studies showed that gold nanoparticles administration in doses 1250–4000 mg/kg into tumor bearing animals prior X-ray irradiation provides significant tumor suppressing effect [8,9,10]. Gold nanoparticles can be also utilized as contrast medium in CT imaging [11,12,13]. However, high concentration of gold is necessary for precise detection of nanoparticles in CT, that hinders acquisition of biodistribution data in vivo. The MRI modality has higher sensitivity than CT, and also provides more valuable diagnostic data. Combining a dose-enhancing gold component with an MRI contrast component within one nanostructure will form an excellent theranostic material both for radiosensitizing and diagnostic imaging. The most commonly used MRI-enhancing element is gadolinium [14], but iron-based contrast agents were also used in clinical practice [15], and additionally, Mn-based contrast agents were explored in preclinical studies [16].

Though both CT and MRI modalities allow imaging of the internal anatomical structures of a studied object, the obtained diagnostic information is quite different. A CT image is the attenuation map of different structures in the studied object; thus, organs that attenuate X-rays very differently (e.g., bones and soft tissues) can be clearly distinguished in CT images, and vice versa—if two adjacent anatomical structures have similar attenuation properties (e.g., a tumor and muscles), it is difficult to differentiate them from each other without additional tools. An MRI image is the map of protons’ nuclear magnetic relaxation rate, whose value depends on the density of the protons (i.e., hydrogen) in a particular region and on their chemical environment. This allows MRI to distinguish different types of soft tissue, which is a valuable property for diagnostics. However, if the imaged region contains little hydrogen, it cannot be visualized properly. For MRI, these “stealth” matter are mainly air and bones [17,18]. CT and MRI provide rather opposing imaging abilities and well complement each other. Using the diagnostic capabilities of both modalities is especially important for oncology, in which tumor growth provides a new and unpredictable anatomical structure (a tumor node) and can cause changes in other anatomical structures, making them difficult to recognize [19,20,21].

We have recently developed novel nanoparticles comprised of gold and iron oxide (Fe_3_O_4_@Au). Gold was chosen as an already proven radioenhancer and CT contrast agent, while iron oxide was chosen as an MRI contrast agent [22]. Fe_3_O_4_@Au nanoparticles can serve as a radiosensitizer in experimental oncology with a prospective translation into clinical oncology. We showed that these nanoparticles in solution served as efficient contrast materials for CT and MRI, even at low concentrations, which are more favorable for clinical use.

Going beyond this early study, here we investigated the in vivo bimodal contrast enhancement ability of the developed nanoparticles, assessed their safety and dosage, and evaluated possible scientific and clinical applications. These studies will pave the way for further investigations of therapeutic efficacy in combination with radiotherapy.

## 2. Results

**Figure 1:**
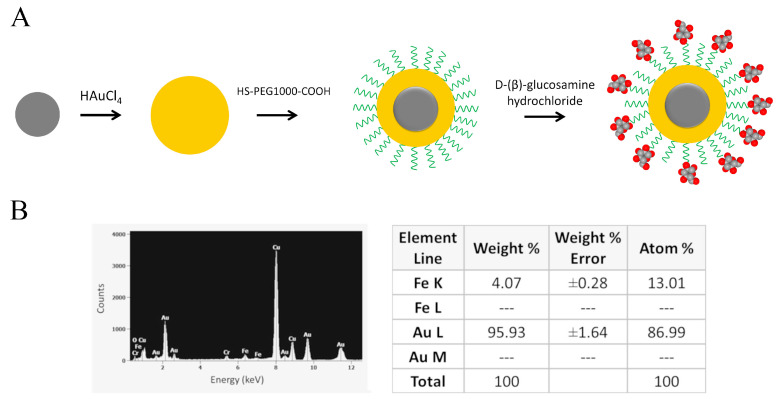
Nanoparticle characterization. (**A**) Scheme of glucose-coated Fe_2_O_4_@Au nanoparticles. Iron oxide nanoparticles were coated with a gold shell and then with a PEG1000 linker for subsequent glucose conjugation; (**B**) EDAX spectrum profile showed absorption peaks for Au and Fe in Fe_2_O_4_@Au particles (**left**), and quantification of the EDAX results is shown (table, **right**).

Fe_3_O_4_@Au nanoparticles were produced in two stages: first, iron oxide nanoparticles were synthesized, and then a gold shell was formed upon them. For the synthesis of core-shell Fe_3_O_4_@Au nanoparticles (Figure 1A), iron oxide nanoparticles were first prepared using a mixture of ferric chloride hexahydrate and ferrous chloride tetrahydrate and dextran coating to prevent aggregation (as described in Methods); transmission electron microscopy (TEM) indicated uniformly distributed spherical Fe_3_O_4_ nanoparticles with a mean diameter of 8 ± 2 nm. To form a gold shell, the dextran coating was removed and replaced by a citrate layer, and the iron oxide nanoparticle solution was then added to an Au solution (50% *w/v* HAuCl_4_), yielding core-shell Fe_3_O_4_@Au nanoparticles. Next, a SH-PEG-COOH linker was added, and the core-shell nanoparticles were then coated with glucose (Methods), as we have previously shown that glucose coating can enhance tumor uptake [23,24,25].

**Figure 2:**
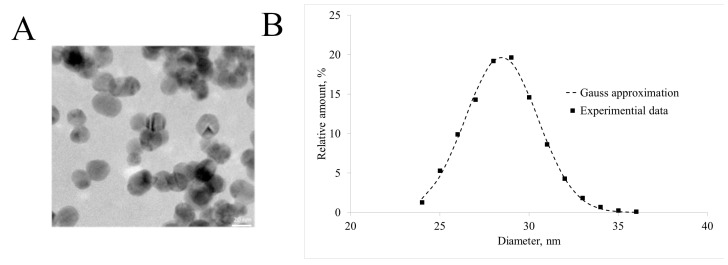
Nanoparticle characterization. (**A**) TEM image of the final nanoparticles. Scale bar = 20 nm; (**B**) the hydrodynamic diameter Fe_3_O_4_@Au NPs distribution and its approximation by the Gauss function.

UV-vis spectra showed a surface plasmon resonance peak at 522 nm. EDAX analysis of the nanoparticle composition validated the presence of both Fe and Au in the final particle, showing average weight percentages of 4.07% for Fe and 95.93% for Au (Figure 1B). TEM imaging of the Fe_3_O_4_@Au nanoparticles showed that uniform spherical nanoparticles, sized 27 nm, were obtained (Figure 2A). The mean hydrodynamic diameter, according to DLS, was 28.5 nm, SD = 2.0 nm (Figure 2B).

To study the in vivo contrast-enhancing efficacy of the bimodal nanoparticles, CT and MRI imaging of Ca755 tumor-bearing mice were performed before and after intravenous Fe_3_O_4_@Au injection (at several timepoints from 5 min up to 17 days post injection).

The nanoparticles caused significant enhancement of blood vessels, both on CT and MRI images. Even medium-sized blood vessels were clearly distinguished (Figure S1). Heart chambers were clearly visualized with CT (Figure S2). The cardiovascular system remained enhanced from 5 min to 90 min after administration. Significant lung edema was observed in the CT images at 22 min after injection: the radiodensity of pulmonary tissue increased and was similar to that of soft tissue. The edema gradually decreased by 90 min post-injection and was completely gone by day 17 (Figure S3).

**Figure 3:**
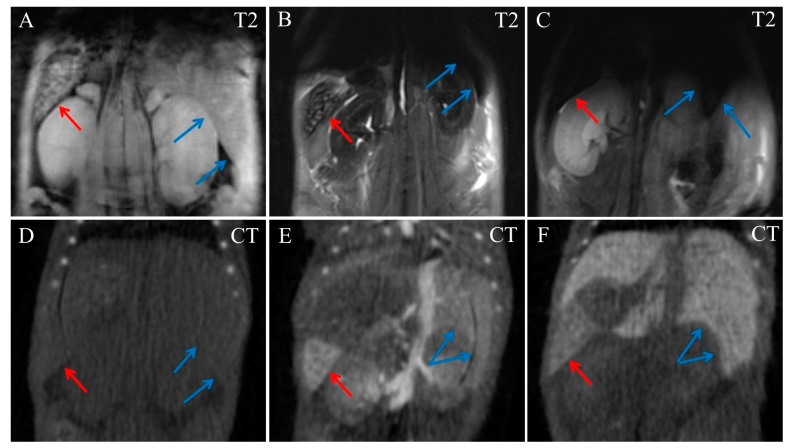
Coronal MRI and CT images of the mouse abdominal area. (**A**) Native T2 MRI; (**B**) T2 MRI, 5 min post injection of Fe_3_O_4_@Au; (**C**) T2 MRI, 17 days post injection of Fe_3_O_4_@Au; (**D**) Native CT image; (**E**) CT image, 22 min post injection of Fe_3_O_4_@Au; (**F**) CT image, 17 days post injection of Fe_3_O_4_@Au. Red arrows indicate the spleen, blue arrows indicate margin of the liver.

Liver tissues became almost homogenously dark in MRI images even at 5 min post injection (Figure 3B), which did not allow us to distinguish its vessels. In contrast, at 22 min post injection, liver blood vessels were clearly visible in CT images, with the liver tissue itself slightly enhanced (Figure 3E). Liver parenchyma radiodensity increased gradually, reaching its maximum by day 17 post-injection, while the contrast agent had already cleared from its blood vessels (Figure 3F).

The spleen also accumulated nanoparticles avidly and was highly hyperdense in CT images and hypointense in MRI, from 5 min to 17 days post-injection (Figure 3). At day 17, gold was observed only in the spleen and the liver, and traces of gold were detected in the kidneys’ parenchyma. Other organs, including the cardiovascular system, returned to baseline completely at this timepoint.

**Figure 4:**
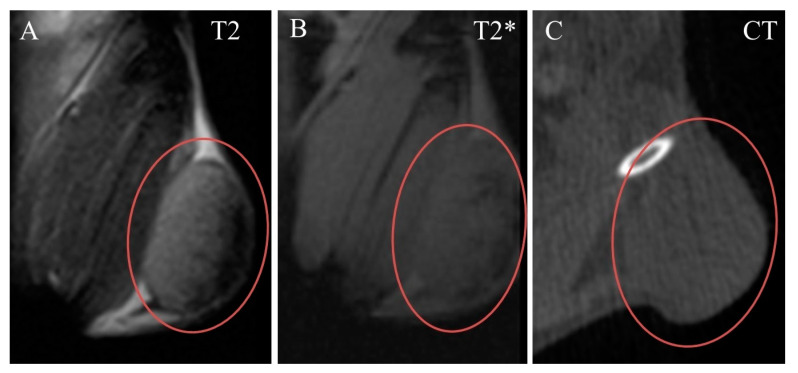
Native coronal images of Ca755 mammary carcinoma. (**A**) T2 MRI; (**B**) T2* MRI; (**C**) CT image. The tumor is located on the right hind leg.

The tumor was clearly seen in CT and MRI. It was nearly ellipse-shaped and approximately 7 × 7 × 5 mm in size. Native images of the tumor were slightly hyperintense in T2-weighted, and nearly isointense in T2*-weighted MRI, compared to adjacent muscle (Figure 4A,B). Its structure was nearly homogenous, with a small subcapsular T2-hypointense portion (Figure 4A). In CT, the tumor was isodense to muscle and completely homogenous (Figure 4C).

**Figure 5:**
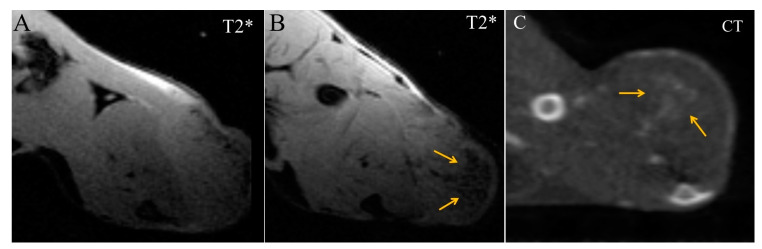
Transversal images of Ca755 mammary carcinoma. (**A**) native T2* MRI; (**B**) T2* MRI 16 min post injection of Fe_3_O_4_@Au; (**C**) CT image 22 min post injection of Fe_3_O_4_@Au. Arrows mark a triangular-shaped area of accumulation.

Starting from 16 min after Fe_3_O_4_@Au administration, diffuse regions of moderate enhancement appeared in the CT and MRI images of the tumor (Figure 5). They were T2*-hypointense in MRI scans but could be more precisely visualized in CT images as a triangular-shaped hyperdense zone.

**Figure 6:**
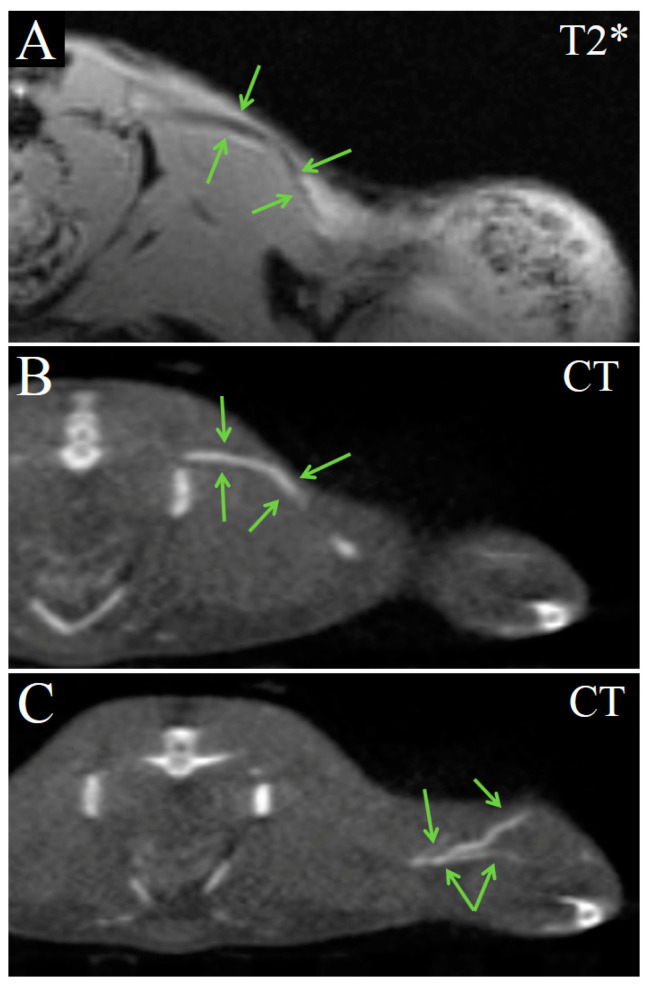
Transversal images demonstrate the enhancement of blood vessels. (**A**) T2* MRI 16 min post injection of Fe_3_O_4_@Au; (**B**) CT image 22 min post injection of Fe_3_O_4_@Au; (**C**) CT image of the tumor 22 min post injection of Fe_3_O_4_@Au. Arrows indicate blood vessels.

Tumor blood vessels were enhanced and clearly distinguished in both imaging modalities (Figure 6). The enhancement was seen not only in large nutrient vessels but also in small convoluted vessels inside the tumor. CT allowed more detailed imaging of tumor vasculature using the Fe_3_O_4_@Au bimodal nanoparticles.

**Figure 7:**
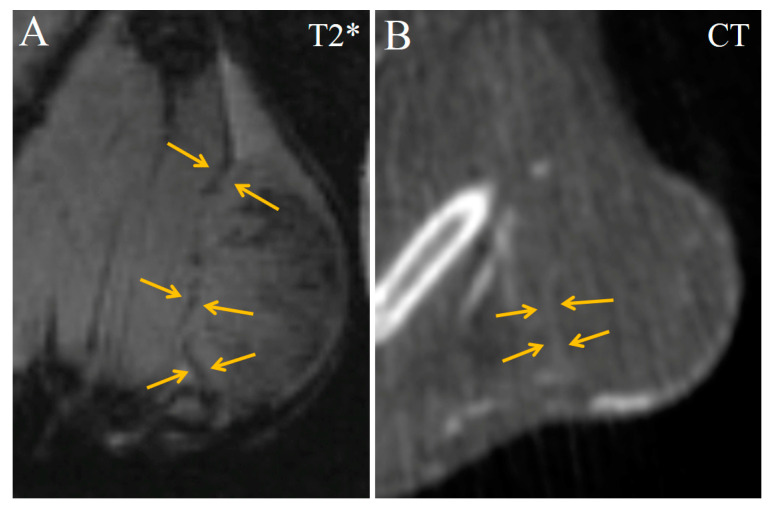
Coronal images of Ca755 mammary carcinoma. (**A**) T2*MRI 16 min post injection of Fe_3_O_4_@Au; (**B**) CT image 22 min post injection of Fe_3_O_4_@Au. Enhanced lines in the inner periphery (related to the tumor capsule) distinguish the tumor from adjacent muscle.

Enhanced lines in the inner periphery, related to the tumor capsule, vaguely demarcated the tumor from the adjacent muscles (Figure 7). The subcutaneous part of the tumor capsule avidly accumulated contrast agent, and became strongly hyperdense in CT (Figure 7B).

**Figure 8:**
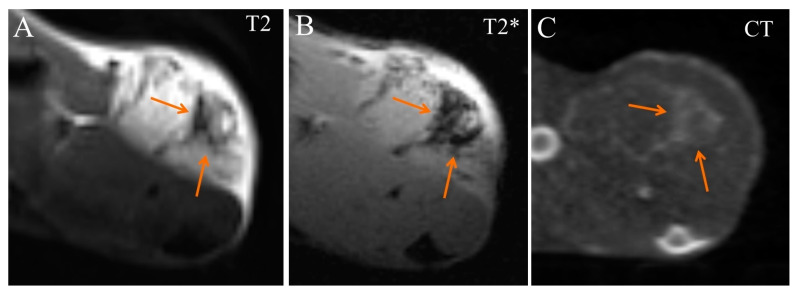
Transversal images of Ca755 mammary carcinoma. (**A**) T2 MRI 50 min post injection of Fe_3_O_4_@Au; (**B**) T2* MRI 50 min post injection of Fe_3_O_4_@Au; (**C**) CT image 65 min post injection of Fe_3_O_4_@Au. Arrows mark the enhanced area of the tumor.

Starting from 50 min after injection, the contrast-enhanced region enlarged and occupied most of the tumor volume (Figure 8). This indicates that more nanoparticles extravasated and accumulated in the tumor tissue. Enhanced areas became more hypointense in T2*-weighted MRI images and more hyperdense in CT images (Figure 8).

**Figure 9:**
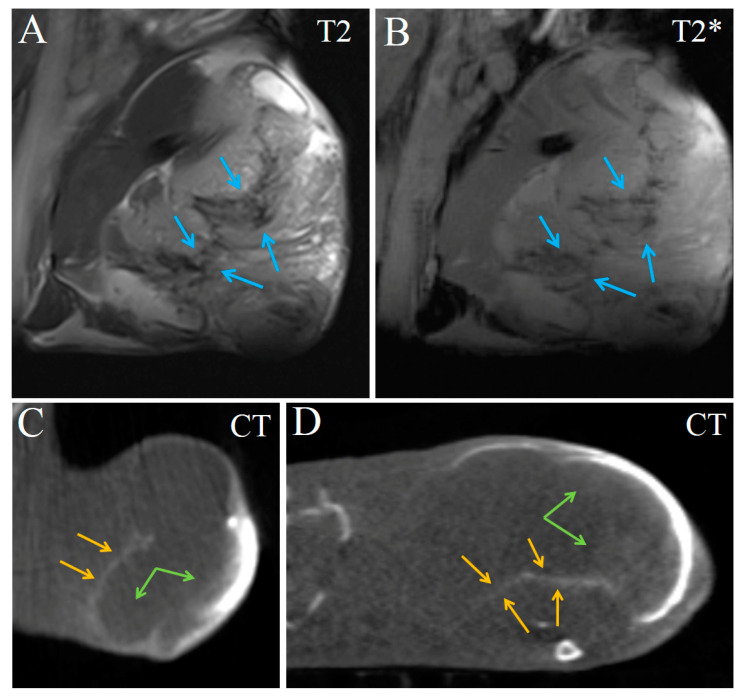
Images of Ca755 mammary carcinoma at 17 days post injection of Fe_3_O_4_@Au. (**A**) T2 MRI, coronal image; (**B**) T2* MRI, coronal image. Blue arrows mark necrotic regions; (**C**) CT, coronal image; (**D**) CT, transversal image. Orange arrows indicate the connective tissue septa, green arrows indicate the area of nanoparticle accumulation.

On the 17th day post injection, the tumor volume significantly increased and contained necrotic areas, which were hypointense in T2- and T2*-weighted MRI and could hardly be distinguished from contrast-enhanced areas (Figure 9A,B). In CT images, the tumor’s structure remained homogenous, and areas of nanoparticle accumulation were easier to recognize (Figure 9B,C). Nanoparticles were located mostly in connective tissue septa and tumor capsules. Most likely, such a specific pattern formed due to the absorption of the nanoparticles by tumor-associated macrophages, with the subsequent migration of these cells to the capsule and accumulation within. Contrast-enhanced areas in the tumor became enlarged, but only minimally hyperdense and vague, without precise margins. The decrease in tumor tissue’s radiodensity was possibly related to enlargement of the tumor volume and, thus, to a decrease in nanoparticle concentration. Blood vessels did not contain any contrast agent at 17 days, all over the mouse body.

## 3. Discussion

We have evaluated core/shell Fe_3_O_4_@Au as a contrast material for CT and MRI modalities in vivo. Although investigation of radiosensitizing properties was not performed in this study, we believe that nanoparticles can provide sufficient therapeutic efficacy in combination with radiotherapy due to their high gold content and suitable particle size. The surface of nanoparticles was formed by a thick gold shell, so from a physical and physiological point of view, they are similar to gold nanoparticles, whose therapeutic efficacy has been proven by numerous studies [26,27,28].

Biodistribution data is essential for the application of theranostic nanoparticles in radiotherapy. Precise definition of nanoparticle uptake regions and their assignment to either tumor node or surrounding normal tissue is necessary for correct treatment planning. Since theranostic nanoparticles can serve as contrast enhancement agents, diagnostic imaging modalities can be used to acquire a biodistribution map in vivo.

The limitations of CT in imaging different soft tissues become an advantage in the case of nanoparticle mapping. The inherent uniformity of soft tissue representation in CT images does not interfere with the contrast agent pattern perception and allows better imaging of the contrast agent distribution. In contrast, for MRI, various specific physiological and morphological peculiarities, as well as imaging artifacts, can interfere with and even hide the contrast agent distribution, both positive and negative [29,30]. For example, necrosis and hemorrhage areas can mask negative contrast agent patterns, and soft tissue edema as well as marginal magnetic field artifacts can mask positive contrast agent distribution [31,32].

The correct assignment of a theranostic drug or contrast agent to a corresponding anatomical structure is sophisticated for imaging tumors, whose shape and borders are often difficult to identify with CT imaging. Using the bimodal Fe_3_O_4_@Au, enhanced areas around the tumor can be clearly seen; however, it was difficult to identify whether this area was part of the tumor volume or the surrounding normal tissues. Corresponding T2- and T2*-weighted images revealed that the enhanced areas belong to the tumor node, and significant enhancement was seen both at the tumor’s border and in some areas within the tumor (Figure 8).

Sun et al. applied 75 nm micelles, simultaneously loaded with 1.9 nm Au and 15 nm Fe_3_O_4_ nanoparticles, for contrast enhancement of orthotopic and subcutaneous U251 gliomas in CT and MRI modalities. The tumor was sufficiently enhanced in T2 MRI, but CT images demonstrated no contrast enhancement, probably due to the low amount of gold accumulated in the tumor tissue. The radiosensitizing potential of developed micelles was demonstrated in vitro [33].

Goubault et al. developed radiosensitizing Fe-Au hollow nanocapsules (~100 nm) with a hybrid shell made of crosslinked polymers and nanoparticles. A survival in vivo study was performed to evaluate the therapeutic effect of radiotherapy in combination with Fe-Au nanocapsules. Irradiation was applied after intratumoral injection of nanocapsules to mice with orthotopic GL261 gliomas. The group of mice treated with Fe-Au nanocapsules combined with irradiation exhibited longer median survival (28 days), compared with the irradiated-only group (24 days). Tumor growth was also monitored in MRI. Nanocapsules were clearly visualized in an MRI, and their long retention in the tumor tissue was confirmed [34].

However, using micelles for radiosensitization could be questionable because of their possible instability in vivo. Particularly, cross-linking polymers can be disrupted by enzymes. Core/shell nanoparticles seem to be a better option.

Kang et al. synthesized nanoflower-like core/shell Fe_3_O_4_/Au nanoparticles and used them for bimodal MRI and photoacoustic imaging. They observed significant enhancement of the tumor in T2-weighted MRI after intravenous injection of mice with LNCaP xenograft, especially with magnetic targeting. Computed tomography was not performed in this study. Nanoparticles also provided a sufficient signal in photoacoustic imaging, but this method is not widely used in clinical practice [35].

Core-shell Fe@Au nanostructures were investigated as a theranostic agent for photothermal therapy. Caro et al. studied the biodistribution of core-shell Fe@Au nanoparticles coated with polyvinylpyrrolidone in mice with subcutaneous C6 tumors. After intravenous injection, nanoparticles had a blood circulation time below 24 h, and could not effectively accumulate in the tumor. So photothermal therapy was performed after intratumoral injection [36]. Similar results were obtained by Li et al.: only intratumoral injection provided a sufficient concentration of Fe_3_O_4_@Au core/shell nanostars in the subcutaneous HeLa xenograft [37]. Despite the promising results of photothermal therapy in vivo, the lack of tumor targeting after intravenous injection may be an obstacle to clinical translation.

Griaznova et al. reported successful tumor targeting of laser-ablated Fe-Au core-satellite nanoparticles. Nanoparticles were injected intravenously into mice with EMT6/P carcinoma. An ex vivo biodistribution study revealed that the concentration of nanoparticles in the tumor reached 17 ± 5% ID/g. In vivo, a significant decrease in signal was detected in T2-weighted MRI. However, no contrast enhancement was observed in CT images, perhaps due to the rather low dose of injected gold: a total of 1 mg of Fe-Au@PAA was injected into a mouse [38].

Fe_3_O_4_@Au nanoparticles used in the current study are suggested to serve as a theranostic dose-enhancing agent for radiotherapy, which requires high gold concentration in the tumor. Thus, 0.72 mg of iron and 30 mg of gold were administered intravenously to each mouse, and significant contrast enhancement of the tumor (subcutaneous Ca755 carcinoma) was observed. Moreover, colocalization of enhanced region in MRI and CT modalities was shown. The concentration of Fe_3_O_4_@Au nanoparticles in the tumor tissue was high enough for radiotherapy up to 17 days post injection. That makes Fe_3_O_4_@Au nanoparticles a promising substance for theranostic application in oncology.

In our study, we also observed that Fe_3_O_4_@Au nanoparticles were absorbed by the liver and spleen and retained for at least 17 days with no signs of excretion from the organism. Long-term retention of nanoparticles in the organism raises the issue of their possible biodegradation and loss of MRI properties. As Zelepukin et al. reported, magnetic iron oxide nanoparticles can slowly degrade in the mouse body. The biotransformation half-life varied from 6.8 to 430 days depending on the external polymer coating of the iron oxide core [39]. Kolosnjaj-Tabi et al. also observed that the iron oxide moiety of gold/iron oxide nanoheterostructures dissolved in 14 days after intravenous injection into mice [40]. However, the mentioned nanoheterostructures consisted of a gold core and an iron oxide coating, so iron oxide was outside and exposed to lysosomal enzymes and an acidic environment. In our Fe_3_O_4_@Au nanoparticles, the iron oxide core is inside the thick gold shell. We suppose that they are highly stable in physiological conditions because metal gold is chemically inert and cannot be broken down by lysosomal enzymes. An indirect evidence of stability was persistent contrast enhancement of the spleen, liver, and tumor capsule in at least 17 days both in CT and MRI modalities, without any decrease in signal.

Despite the retention of nanoparticles in the liver and spleen, mice exhibited no signs of toxicity within 17 days. All mice continued to gain normal amounts of body mass, and no changes were observed in their water and food consumption or in their behavior. In CT images, no pathological changes of the liver were detected: the liver had smooth margins and a homogenous structure, and the size of the organ was not enlarged within 17 days post-injection. We could not evaluate long-term toxicity because only tumor-bearing mice were used in this study, so all mice were sacrificed within 17 days in accordance with ethical standards.

Besides theranostics, our bimodal Fe_3_O_4_@Au nanoparticles also have potential use for clinical interventional treatments in oncology, such as different types of tumor ablation and embolization, which require blood vessel enhancement for X-ray and MRI modalities lasting up to several hours. A single dose of Fe_3_O_4_@Au can likely provide better enhancement of images and be better tolerated by patients than multiple injections of iodine and gadolinium contrast agents during the same medical procedure.

Fe_3_O_4_@Au can also aid animal research, as it allows enhanced imaging with MRI and CT in the same animal, with a single contrast medium and single injection, thus obtaining maximum information with a minimum number of animals and manipulations, making animal imaging studies more reliable, informative, and ethical, and at the same time, less tedious.

## 4. Materials and Methods

### 4.1. Nanoparticle Synthesis

Fe_3_O_4_@Au synthesis was described in detail elsewhere [22]. Briefly, dextran T1-coated iron oxide nanoparticles were prepared by a mixture of ferric chloride hexahydrate (3 g) and ferrous chloride tetrahydrate (1.5 g) in 32 mL deionized water mixed with dextran T1 (Mw = 41 kDa) solution (1.7 g in 20 mL deionized water) for 30 min at room temperature under nitrogen flow. The mixture was cooled to 5 °C, and ammonium hydroxide (28%, 12.7 g) was added under stirring for 2 min. Then, the mixture was heated to 60 °C for 40 min, and to 80 °C for 2 h. To allow coating of the particles with a gold shell, the dextran coating was removed and replaced by a citrate layer by washing the solid phase twice with a 10% sodium citrate solution followed by centrifugation (4000 rpm, 20 min). After purification, the iron oxide particle pellet was dissolved in 10% sodium citrate solution to yield a final Fe concentration of 4 mg/mL. To form a gold shell, an Au solution (414 μL of 50% *w/v* HAuCl_4_ in 200 mL of purified water) was heated until boiling, and 4.04 mL of the as-prepared IONP solution were added under stirring (10 min), yielding core/shell Fe_3_O_4_@Au nanoparticles. After cooling to room temperature, the SH-PEG-COOH (1 kDa) solution (80 µL, 36.5 mg/mL) was added and stirred for 3 h. Next, the particles were coated by D-(β)-glucosamine hydrochloride (30 µL, 25 mg/mL), which was added to the solution together with *N*-ethyl-*N*-(3-dimethylaminopropyl) carbodiimide (EDC, 200 µL, 10 mg/mL) and *N*-hydroxysuccinimide (NHS, 200 µL, 10 mg/mL), followed by 3 h of stirring at room temperature.

The determination of iron and gold concentrations in the final solution was carried out by inductively coupled plasma (ICP) atomic emission spectrometry (Agilent 4200 MP-AES, Santa Clara, CA, USA) using calibration curves for each element. Standard solutions with Fe or Au concentrations of 500, 1000, 1500, and 2000 ppb were prepared by dilution of Fe or Au standard solutions (1 mg/mL) in 2% (*w*/*w*) HNO_3_ for ICP (Merck, Rahway, NJ, USA). A 2% aqueous solution of HNO_3_ was also used as a blank. Samples of nanoparticles were prepared by dissolving them (10 μL) with 90 μL of aqua regia (1 mL of HNO_3_ + 3 mL of HCl) at 60 °C for 2 h and then at room temperature for 12 h. After digestion, the solutions were diluted 20 and 450 times for the ICP-AES analysis of iron and gold, respectively. The gold and iron concentrations in the studied solution, determined by ICP-AES, was 164.0 ± 4.0 mg/mL and 4.0 ± 0.1 mg/mL, respectively. Nanoparticles were further characterized by dynamic light scattering (ZetaSizer 3000HS; Malvern Instruments, Malvern, UK), energy-dispersive X-ray spectroscopy, and transmission electron microscopy (TEM, JEM-1400, JEOL, Akishima, Japan).

### 4.2. Animal In Vivo Imaging

Imaging was performed in female C57Bl/6 mice, 20–22 g body weight, with subcutaneous Ca755 mammary adenocarcinoma inoculated into the hind right leg, performed with 0.2 mL of 14% tumor tissue suspension prepared ex tempore. Imaging was conducted when the tumor node volume reached approximately 200 mm^3^. Imaging was performed under isoflurane gas anesthesia. CT imaging of the animals was performed with the IVIS Spectrum CT imaging system (Perkin Elmer, Waltham, MA, USA) and MRI with the ClinScan 7T scanner (Bruker, Billerica, MA, USA). Mice under isoflurane gas anesthesia were injected intravenously via the tail vein with 180 µL of Fe_3_O_4_@Au solution containing 0.72 mg of iron and 30 mg of gold. T2* weighted MRI images of the mice were acquired using gradient echo sequence with the following parameters: TR = 400 ms, TE = 3.5 ms, flip angle = 30, FOV = 27 × 35 mm, base resolution = 200 × 256, slice thickness = 0.69 mm. Each mouse underwent CT and MRI imaging before the Fe_3_O_4_@Au injection and after the injection, at several time points on the day of the injection and at 17 days post-injection. As it was crucial to perform imaging of the same animal with both modalities, MRI and CT scans were made sequentially, at close but different time points. The following MRI and CT timepoint pairs were used for the purpose of comparison: 16 min and 22 min; 50 min and 65 min. Additionally, the following studies, which were not accompanied by another modality, were conducted: MRI, 5 min p.i., and CT, 90 min p.i. The last time point was 17 days post-injection for both modalities. Mice were euthanized on day 17th post Fe_3_O_4_@Au administration, immediately after imaging. All animal studies were performed in accordance with local ethical regulations and approved by the institutional ethics committee.

## 5. Conclusions

Our results showed that bimodal CT-MRI nanoparticles serve as effective contrast agents, providing useful information for tumor diagnostics. CT imaging demonstrated excellent distribution of the bimodal nanoparticle contrast agent, while MRI allowed reliable identification of the particular organs and structures to which the obtained distribution belonged. Application of bimodal Fe_3_O_4_@Au as contrast agents for MRI/CT is especially valuable for tumor study and imaging. The unpredictability and variety of tumors’ shape and location require special imaging tools for proper tumor diagnostics and treatment.

Due to their high gold content and long retention in tumors, theranostic Fe_3_O_4_@Au nanoparticles can be considered promising radiosensitizers for radiotherapy. Although the uptake and retention of the Fe_3_O_4_@Au nanoparticles in the liver could limit routine clinical application of the nanoparticles, their use for imaging and therapy in cancer patients can be highly beneficial in oncological patients, especially with advanced tumors with a poor prognosis.

## Data Availability

Data is contained within the article.

## Supplementary Materials

The following supporting information can be downloaded at: https://www.mdpi.com/article/10.3390/ijms24010070/s1. Figure S1: Enhancement of blood vessels; Figure S2: Enhancement of heart chambers; Figure S3: Transversal CT images of mouse lungs.
