# Supplementary material for: CT and MRI Imaging of Theranostic Bimodal Fe3O4@Au NanoParticles in Tumor Bearing Mice
Source: Int J Mol Sci. 2022 Dec 21;24(1):70. doi: 10.3390/ijms24010070 (PMC9820463; doi:10.3390/ijms24010070)
Supplement: Supplementary file 1 [file ijms-24-00070-s001.zip › ijms-2086694-supplementary.pdf]

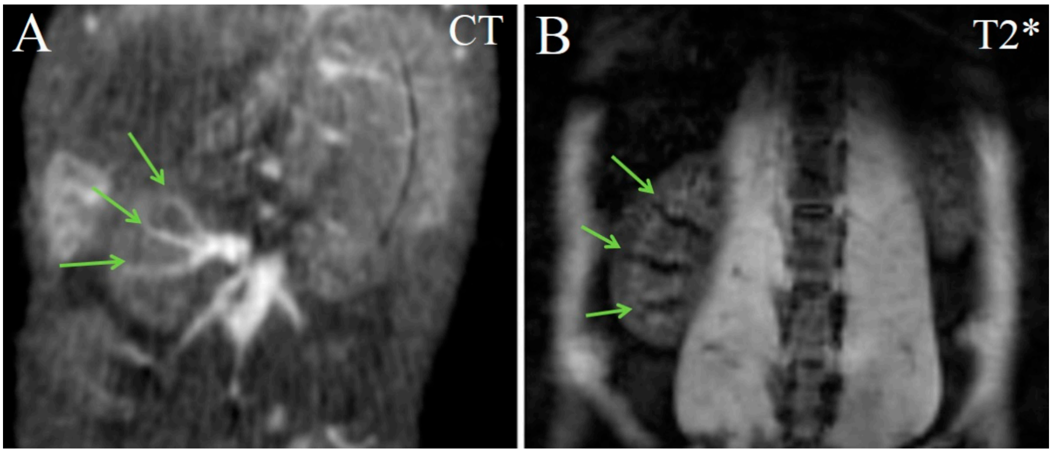

Figure S1. Enhancement of blood vessels. (**A**) Coronal CT image 22 min post injection of Fe_3O_4@Au; (**B**) Coronal T2* MRI 16 min post injection of Fe_3O_4@Au. Arrows mark blood vessels of the kidney.

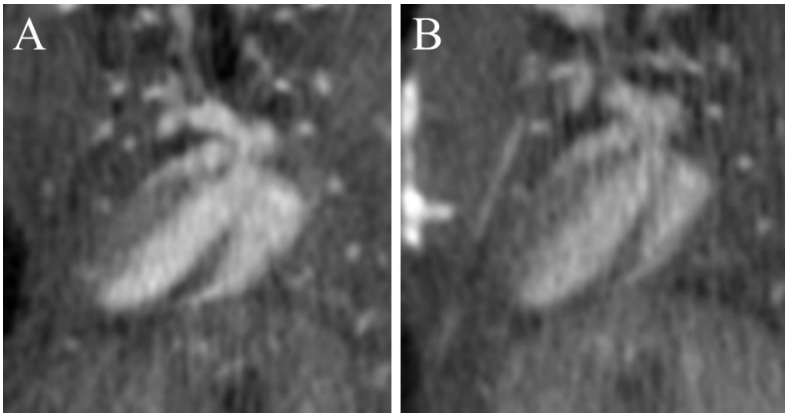

Figure S2. Enhancement of heart chambers. Coronal CT image. (**A**) CT image 22 min post injection of Fe_3O_4@Au; (B) CT image 90 min post injection of Fe_3O_4@Au.

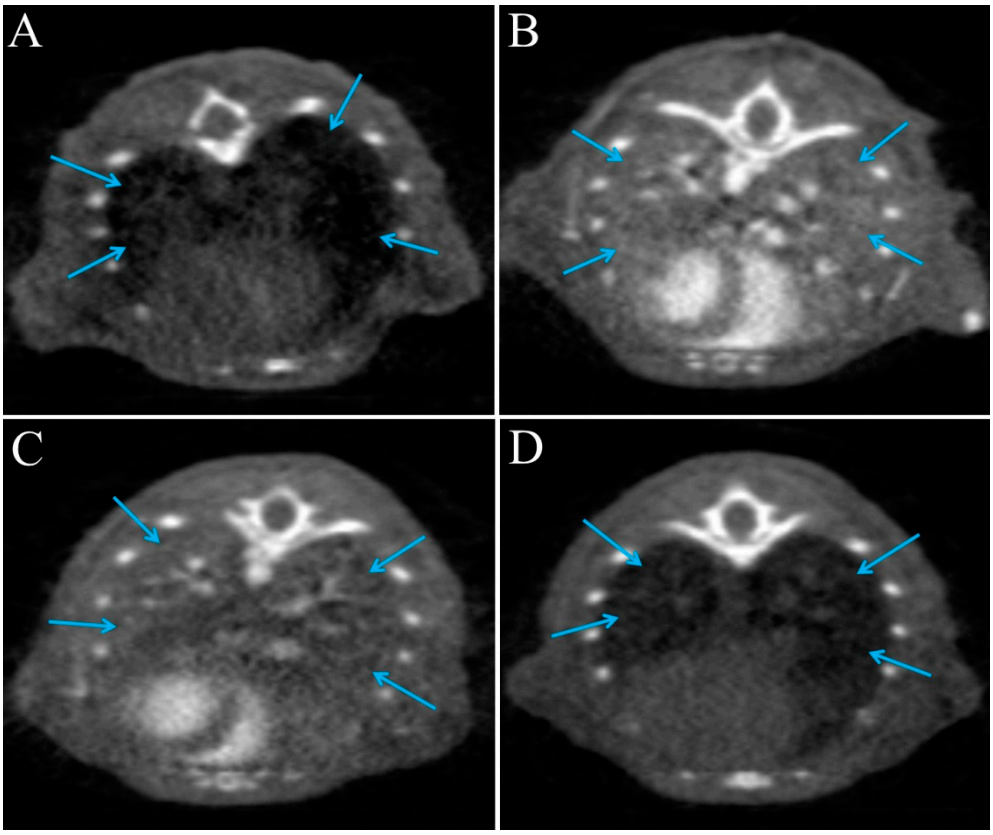

Figure S3. Transversal CT images of mouse lungs. (**A**) Native CT image; (**B**) CT image 22 min post injection of Fe_3O_4@Au; (**C**) CT image 90 min post injection of Fe_3O_4@Au; (**D**) CT image 17 days post injection of Fe_3O_4@Au. Arrows mark pulmonary tissue.
